# Maintaining therapeutic continuity in adolescent psychiatric day hospital programs during the COVID-19 lockdown

**DOI:** 10.1192/j.eurpsy.2021.276

**Published:** 2021-08-13

**Authors:** G. De Vita, A. Terrinoni, F. Di Santo, D. Calderoni, E. Rainò, A. Anichini, M. Ferrara

**Affiliations:** 1 Department Of Human Neuroscience, Section Of Child And Adolescent Neuropsychiatry, Sapienza University of Rome, Rome, Italy; 2 Department Of Public Health And Pediatric, Division Of Child Neurology And Psychiatrysciences, University of Turin, Turin, Italy

**Keywords:** COVID-19, telemedicine, Adolescence Health Care Services, IES-R

## Abstract

**Introduction:**

The COVID-19 social lockdown imposed important limitation to non-emergency health care services in Italy, between March and May 2020, with many difficulties in the mental health assistance of those chronic conditions needing a continuative therapeutic support.

**Objectives:**

Our study aimed to describe how therapeutic activities have been carried on by remote services in two Adolescent Psychiatric Day Hospital Units (Rome and Turin) and the outcome of these assistance interventions in youths with subacute psychopathology.

**Methods:**

The patient cohort includes 162 adolescents (12-19 years old; QI>70) DH outpatients presenting a complete clinical and neuropsychiatric assessment before the lockdown. During the several phases of COVID-19 quarantine all patients were monitored and supported by telemedicine interventions. All data were recorded and standardized every 15 days: symptom severity was rated by global severity (CGI-S) and stress level by self-reported measures of stress (IES-R).

**Results:**

Among patients, CGI score remained stable, IES-R score declined over time: higher IES-R score was significantly associated with female gender and but no differences was observed related with the primary diagnosis. 5 patients presented a clinical acute state needing a hospitalization. The rate of hospitalization was not significantly different compared with the rate observed in the same period of 2019.

**Conclusions:**

In youth with psychopathological conditions, remote assistance for psychiatric cares resulted effective and it was associated with a clinical stability with decreasing stress levels.

**Disclosure:**

No significant relationships.

